# Prospective associations between problematic eating attitudes in midchildhood and the future onset of adolescent obesity and high blood pressure^1^,^2^,^3^

**DOI:** 10.3945/ajcn.116.141697

**Published:** 2016-12-14

**Authors:** Kaitlin H Wade, Michael S Kramer, Emily Oken, Nicholas J Timpson, Oleg Skugarevsky, Rita Patel, Natalia Bogdanovich, Konstantin Vilchuck, George Davey Smith, Jennifer Thompson, Richard M Martin

**Affiliations:** 4 School of Social and Community Medicine, Faculty of Health Sciences,; 5 Medical Research Council Integrative Epidemiology Unit, and; 6 University Hospitals Bristol National Health Service Foundation Trust, National Institute for Health Research Bristol Nutrition Biomedical Research Unit, University of Bristol, Bristol, United Kingdom;; 7 Departments of Pediatrics and; 8 Epidemiology, Biostatistics and Occupational Health, McGill University Faculty of Medicine, Montreal, Quebec, Canada;; 9 Department of Population Medicine, Harvard Medical School and Harvard Pilgrim Health Care Institute, Boston, MA;; 10 Department of Psychiatry and Medical Psychology, Belarusian State Medical University, Minsk, Belarus; and; 11 National Research and Applied Medicine Mother and Child Centre, Minsk, Belarus

**Keywords:** problematic eating attitudes, blood pressure, adiposity, prospective, adolescents

## Abstract

**Background:** Clinically diagnosed eating disorders may have adverse cardiometabolic consequences, including overweight or obesity and high blood pressure. However, the link between problematic eating attitudes in early adolescence, which can lead to disordered eating behaviors, and future cardiometabolic health is, to our knowledge, unknown.

**Objective:** We assessed whether variations in midchildhood eating attitudes influence the future development of overweight or obesity and high blood pressure.

**Design:** Of 17,046 children who participated in the Promotion of Breastfeeding Intervention Trial (PROBIT), we included 13,557 participants (79.5% response rate) who completed the Children’s Eating Attitudes Test (ChEAT) at age 11.5 y and in whom we measured adiposity and blood pressure at ages 6.5, 11.5, and 16 y. We assessed whether ChEAT scores ≥85th percentile (indicative of problematic eating attitudes) compared with scores <85th percentile at age 11.5 y were associated with new-onset overweight, obesity, high systolic blood pressure, or high diastolic blood pressure between midchildhood and early adolescence.

**Results:** After controlling for baseline sociodemographic confounders, we observed positive associations of problematic eating attitudes at age 11.5 y with new-onset obesity (OR: 2.18; 95% CI: 1.58, 3.02), new-onset high systolic blood pressure (OR: 1.34; 95% CI: 1.05, 1.70), and new-onset high diastolic blood pressure (OR: 1.25; 95% CI: 0.99, 1.58) at age 16 y. After further controlling for body mass index at age 6.5 y, problematic eating attitudes remained positively associated with new-onset obesity (OR: 1.80; 95% CI: 1.28, 2.53); however, associations with new-onset high blood pressure were attenuated (OR: 1.14; 95% CI: 0.89, 1.45 and OR: 1.09; 95% CI: 0.86, 1.39 for new-onset systolic and diastolic blood pressure, respectively).

**Conclusions:** Problematic eating attitudes in midchildhood seem to be related to the development of obesity in adolescence, a relatively novel observation with potentially important public health implications for obesity control. PROBIT was registered at clinicaltrials.gov as NCT01561612 and isrctn.com as ISRCTN37687716.

## INTRODUCTION

Dieting, deliberate weight loss, and weight control are relatively common among adolescents, affecting 41–62% of females and 20–31% of males in industrialized or developing countries (1–3). Problematic eating behaviors such as very low–calorie diets, skipping meals, and the use of diet pills, powders, and liquids have been reported among 10–27% of adolescents, with a minority (1–9%) reporting more extreme methods of weight control, such as vomiting, laxatives, and fasting (1). However, the extent to which such problematic eating attitudes in childhood and adolescence influence longer-term health is not clear.

Adolescents and young adults with clinically diagnosed eating disorders, such as anorexia and bulimia nervosa, seem to have reduced cardiovascular function and cardiac complications as a result (4–12), whereas binge-eating disorder is associated with higher blood pressure (BP)^12^ and an increased risk of cardiovascular disease (7, 13, 14). Disordered eating behaviors have also been positively associated with later weight gain (4, 6–9, 13–22); therefore, associations of disordered eating attitudes and behaviors with cardiovascular outcomes may be in part attributable to an association between disordered eating and excess weight gain. In addition, population-level variations in eating attitudes may predict the development of eating disorders (6, 23–27) and thereby influence later cardiometabolic health, including adiposity-related traits such as BP.

It is also possible that excess adiposity leads to disordered eating attitudes and behaviors and that this overweight predicts future weight gain and cardiovascular disease risk (28). Because of the lack of large-scale prospective studies with long follow-up periods investigating these relations, the direction of associations (from adiposity to eating problem compared with eating problem to adiposity) cannot be confidently inferred. With the use of an instrumental variable approach aimed at understanding the causal direction of associations, we have previously found that adiposity as early as age 6.5 y may causally influence future eating attitudes measured at age 11.5 y (28). This direction of association could occur if overweight children develop problematic eating attitudes as they enter the socially sensitive teenage years in an attempt to control weight (29). Thus, observed associations between eating behaviors in midchildhood and later cardiometabolic traits (such as BP, overweight, and obesity) may be attributable to early adiposity that affects later eating attitudes (**Supplemental Figure 1**).

We investigated prospective associations of problematic eating attitudes measured at age 11.5 y with the use of the Children’s Eating Attitudes Test (ChEAT) with both adiposity and BP measured 5 y later among Belarusian children participating in PROBIT (Promotion of Breastfeeding Intervention Trial) (NCT01561612 and ISRCTN37687716).

## METHODS

**FIGURE 1:**
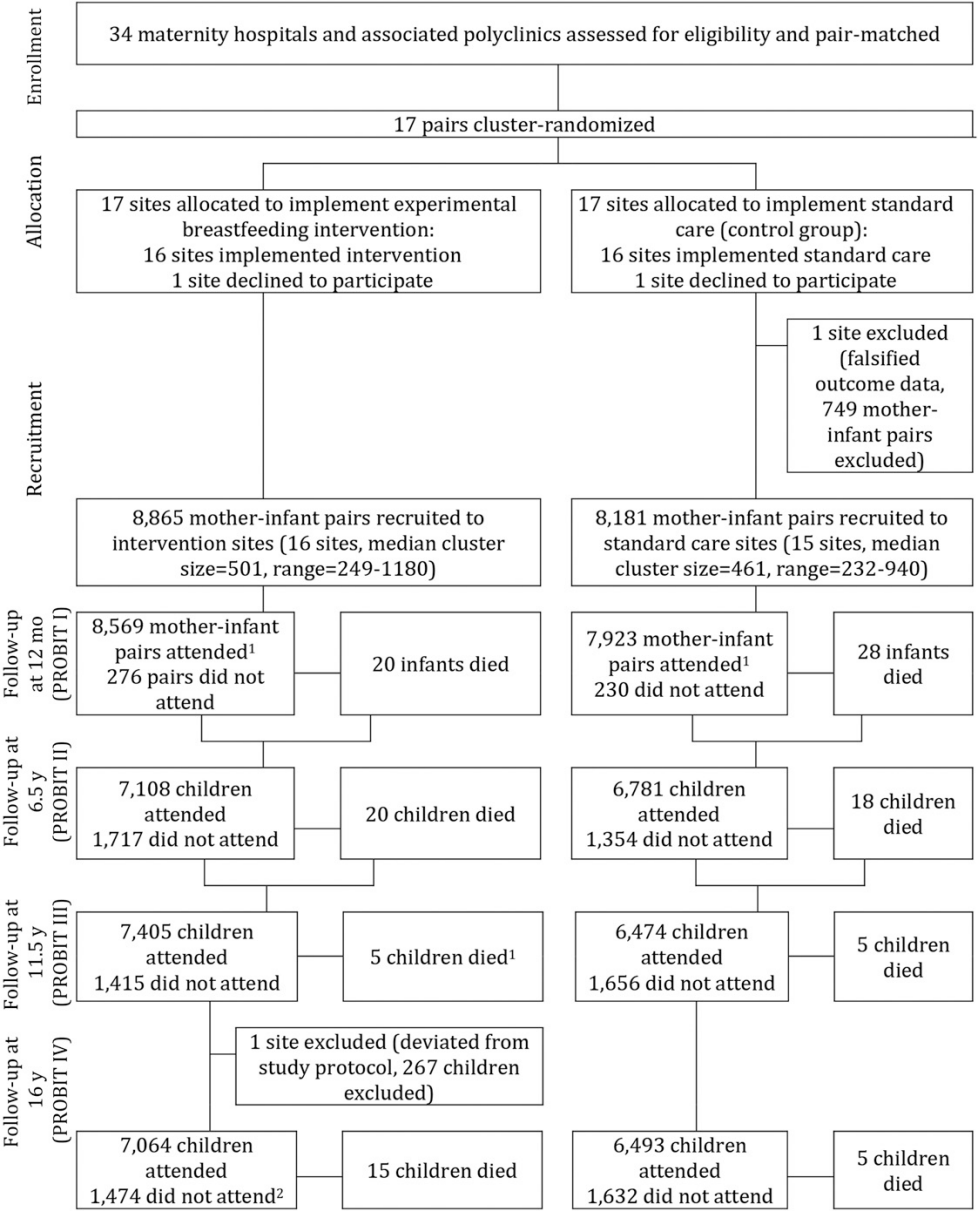
Flow diagram of progress of clusters and individuals through PROBIT recruitment and follow-up phases I–IV in PROBIT. ^1^During PROBIT III, 6 deaths were reported in the intervention arm; data checking during PROBIT IV found 1 of these children had been incorrectly reported as deceased, and data were amended. ^2^Of the 13,557 seen at PROBIT IV, 12,072 were seen at both PROBIT II and III, 274 were not seen at either PROBIT II or III, 449 were seen at PROBIT II but not seen at PROBIT III, and 762 were seen at PROBIT III but not at PROBIT II. PROBIT, Promotion of Breastfeeding Intervention Trial.

We conducted a cohort analysis of children enrolled in PROBIT, a multicenter cluster-randomized controlled trial of an intervention to promote increased breastfeeding duration and exclusivity conducted in Belarus. As detailed previously (30), the trial enrolled 17,046 infants born between 1996 and 1997 from 31 maternity hospitals that were randomly assigned to the intervention (*n* = 16) or control (usual infant feeding practices; *n* = 15) arms. Inclusion criteria specified that infants were full term (≥37 wk of gestation), healthy singletons, and had a birth weight ≥2.5 kg and a 5-min Apgar score ≥5 and that mothers were healthy and had initiated breastfeeding. In total, 16,492 (96.7%) infants were followed up at frequent intervals up to 12 mo of age (specifically at ages 1, 2, 3, 6, 9, and 12 mo), and 13,889 children (81.5%) were followed up at age 6.5 y, 13,879 (81.4%) at age 11.5 y, and 13,557 (79.5%) at age 16 y (**Figure 1**). We excluded 16-y follow-up data from 1 polyclinic because it deviated from study protocol; this excluded 267 mother-infant pairs, leaving 30 eligible polyclinics for this analysis (*n* = 15 in both the experimental and control arms).

### Measurement of eating attitudes

At age 11.5 y, 13,751 children completed a modified version of ChEAT, a questionnaire assessing problematic eating attitudes, including food preoccupation; peer and media pressure about eating, weight, and body image; dieting; purging; and food restriction (23). Our previous methods that used ChEAT have been published previously (28, 31, 32). Briefly, because Maloney et al. (33) found that 1 ChEAT question (“I can show self-control around food”) was negatively correlated with the remainder of the questionnaire, we administered the remaining 25 questions only. After pilot work with Belarusian pediatricians suggested that children would be confused by the multiple response options, we simplified the original 6-item Likert response scale to a 3-item scale: often, sometimes, and never.

In preliminary factor analyses, we found that question 25 (“I enjoy trying new rich foods”) was inversely correlated with the total ChEAT score; therefore, we excluded it from our main analyses. All responses were scored as 3 (often), 1.5 (sometimes), or 0 (never), yielding a range of 0 to 72 for ChEAT-24 scores, a similar range (0–78) as in ChEAT-26 scores. ChEAT is therefore a quantitative indicator of problematic eating attitudes that may be symptomatic of more severe eating disorders (34).

In line with previous studies (23–27, 33, 35) and to limit the number of false positives, we used a ChEAT-24 score ≥22.5, corresponding to the 85th percentile in our data, as a threshold to indicate problematic eating attitudes. In a sensitivity analysis, we investigated associations with the use of a ChEAT-24 score ≥25.5 [91st percentile; comparable to Maloney et al. (33)]. We did not investigate ChEAT-24 scores as a continuous outcome because the scores were positively skewed and had a bimodal distribution (10.1% of children answered never to all questions). In a second sensitivity analysis, question 25 was included and scored in the opposite direction from all other responses, yielding a ChEAT-25 score ranging from 0 to 75. The corresponding 85th and 91st percentile scores were 24 and 27, respectively.

We conducted an audit of 141 randomly selected participating children a mean of 1.3 y (range: 0.2–2.4) after the initial study visit to assess the reproducibility of the scores. Percentage agreement for the original and audit ChEAT-24 scores ≥85th percentile was 85.1% for the 141 children; Cohen’s κ (chance-corrected agreement) was 0.46 (95% CI: 0.30, 0.63), indicating moderate test-retest reproducibility.

### Measurement of outcomes

At the 11.5- and 16-y visits, measures of BP were obtained by pediatricians who attended training workshops following a detailed research protocol. Participants sat at rest for 5 min, after which measures of systolic BP (SBP) and diastolic BP (DBP) were obtained with the use of a fully automated Omron 705IT device. SBP and DBP were measured 3 times at age 11.5 y and twice at age 16 y in the right arm in a seated position with the use of the appropriate cuff size and a 1-min interval between readings. We used the mean of all readings for each child at these visits in all analyses.

Pediatricians measured children’s weight and height at ages 11.5 and 16 y as described previously (36, 37). Standing height was measured twice to the nearest centimeter with the use of a stadiometer with a movable headboard. If the child’s height measurements differed by >0.5 cm, then a third and fourth measurement were taken. The mean of all available measurements was used in all analyses. We also calculated the BMI (in kg/m^2^) for all children. The Tanita TBF 300GS body-fat analyzer was used to measure bioelectrical impedance from foot to foot, which provided measurements of weight, BMI, percentage body fat, fat mass, and fat-free mass. All measures were obtained after the child had urinated (if needed) and removed any heavy items of clothing, shoes, and socks. We calculated the fat mass index (FMI) by dividing fat mass by height as a more accurate measure of adiposity at 16 y.

Measures of SBP, DBP, weight, and height at ages 11.5 and 16 y as well as fat mass at age 16 y that were in excess of ±4 SDs from the mean (241 children) were excluded from the analyses because these values were assumed to be errors, in accordance with previous analyses of PROBIT data (28, 31, 32).

We conducted an audit of 132 randomly selected participating children a mean of 1.2 y (range: 0.02–2.5) after the 16-y visit to assess the reproducibility of the measured outcomes. Correlation coefficients for the original and audit SBP, DBP, BMI, and FMI ranged between 0.56 and 0.90 for the 132 children, indicating moderate test-retest reproducibility.

We categorized children’s weight as normal, overweight, or obese at ages 11.5 and 16 y according to their BMI, defined by the age- and sex-specific models recommended by Wade et al. (28) and Cole et al. (38, 39) with trajectories equivalent at age 18 y to the WHO’s defined BMI thresholds of ≤17 (thinness), >17–25 (normal weight), >25–30 (overweight), and >30 (obesity) at ages 11.5 and 16 y. We then created 2 binary variables to represent new-onset overweight at age 16 y (defined as children who were classified as not overweight or obese at age 11.5 y but were overweight at age 16 y) and new-onset obesity at age 16 y (children who were not obese at age 11.5 y but were obese at age 16 y).

Consistent with the fourth report on the diagnosis, evaluation, and treatment of high BP in children and adolescents (40), we standardized BP measures to age, sex, and height. We then created 2 binary variables to represent children who were nonhypertensive and therefore had an SBP or DBP <95th percentile (defined in the entire cohort) at age 11.5 y but who subsequently developed a high SBP or DBP (≥95th percentile of the entire cohort) at age 16 y (i.e., new-onset high SBP or DBP). Because an a priori decision was made to assess weight gain and increased BP as outcomes, we did not compare individuals who were overweight, obese, or had high BP at age 11.5 y who either remained in that state or lost weight or had reduced BP at age 16 y.

### Measurement of covariables

At recruitment, mothers reported parental education (28) and highest household occupation (28, 41, 42). We used these variables as indicators of socioeconomic position.

### Ethics

PROBIT was approved by the Belarusian Ministry of Health and received ethical approval from the McGill University Health Centre Research Ethics Board, Harvard Pilgrim Health Care Institutional Review Board, and Avon Longitudinal Study of Parents and Children Law and Ethics Committee. A parent or legal guardian provided written informed consent in Russian at enrollment and at follow-up visits, and all children provided written assent at ages 11.5 and 16 y.

### Statistics

Multivariable mixed-effects logistic regression models [*xtmelogit* in Stata version 13 (StataCorp)] were used to assess the association of ChEAT-24 scores with new-onset overweight and obesity and new-onset high SBP and DBP at age 16 y. These models account for the moderate degree of within-polyclinic clustering of ChEAT-24 responses (intraclass correlation coefficient: 0.22) and allow inference at the individual level within polyclinic sites. Analyses were performed with the use of the following cluster-adjusted models: a basic model controlling for age at the outcome measure and sex and a confounder-adjusted model additionally controlling for highest household occupation, parental education, location of the polyclinic, and treatment group. As described earlier, our previous study of BMI at age 6.5 y in relation to ChEAT at age 11.5 y with the use of an instrumental variable approach to improve causal inference provided evidence that adiposity at age 6.5 y may be causally related to future problematic eating attitudes (28). Thus, we considered BMI at age 6.5 y to be a potential confounder of the association of problematic eating attitudes at age 11.5 y with all new-onset outcomes at age 16 y (Supplemental Figure 1). To assess the confounding effect of adiposity in the association between problematic eating attitudes at age 11.5 y and new-onset outcomes, we included a third (final) model that additionally controlled for the child’s BMI at age 6.5 y. We did not include BMI at age 11.5 y (measured in a cross-section with respect to problematic eating attitudes) because of concerns about inducing collider bias by adjusting for a potential mediator (43). First, we were interested in the overall effect of problematic eating attitudes on 16-y outcomes, and because part of the effect is likely to be mediated through BMI at age 11.5 y, the adjusted effect would underestimate the overall effect. Second, adjusting for BMI at age 11.5 y (mediator) could bias the effect of problematic eating attitudes in the presence of unknown or unmeasured confounders (common causes of problematic eating attitudes at 11.5- and 16-y outcomes) (**Supplemental Figure 2**). All analyses were performed with the use of Stata version 13.

### Sensitivity analyses

We did not observe any strong evidence for an interaction by sex in the associations between ChEAT-24 scores and most outcomes (**Supplemental Table 1**); therefore, we combined results for males and females. However, in light of evidence for an interaction by sex with new-onset overweight (*P* values for interaction ranged between 0.01 and 0.10 in confounder-adjusted models) and the known differences in the incidence of problematic eating attitudes between females and males (25, 26), we stratified our analyses by sex, the results of which are presented in Supplemental Table 1. In addition to assessing new-onset outcomes, we assessed associations of ChEAT-24 scores ≥85th percentile measured at age 11.5 y with absolute measures of FMI (a more accurate measure of adiposity) and BP at age 16 y in sensitivity analyses with the use of multivariable mixed-effects linear regression models (*xtmixed* in Stata) with the same models adjusted for age, sex, socioeconomic confounders, and BMI at age 6.5 y as described previously. Finally, we hypothesized that BMI at age 11.5 y could mediate the association between problematic eating attitudes at age 11.5 y and later new-onset outcomes and therefore adjusted for BMI at age 6.5 y as a confounder. However, it is possible that BMI at age 11.5 y was in fact a confounder of the association of interest; therefore, as a final sensitivity analysis, we adjusted for BMI at age 11.5 y rather than at 6.5 y.

## RESULTS

Of the 13,557 (80%) individuals who had data at the 16-y visit, 12,072 (89%) had available data from both the 6.5- and 11.5-y visits (Figure 1). Of children who were not categorized as overweight at age 11.5 y, 7% were overweight at age 16 y and 1% had developed new-onset obesity between ages 11.5 and 16 y (**Table 1**). Of children who did not have either high SBP or DBP (defined as <95th percentile) at age 11.5 y, 4% had either high SBP or DBP at age 16 y. To illustrate the distribution of potential confounders in the data, factors associated with problematic eating attitudes (exposure) or new-onset overweight, obesity, or high BP (outcomes) are shown in **Supplemental Tables 2** and **3**, respectively.

**TABLE 1:** Characteristics of PROBIT participants followed up until age 16 y^1^

Characteristics	Children in sample, *n*	Total sample
At recruitment		
Females	16,778	48.20
Within intervention arm	16,778	51.24
From rural areas	16,778	42.01
From East Belarus	16,778	48.01
Children whose parents were nonmanual workers	15,535	56.40
Mother’s education ≥ university	16,778	13.44
Father’s education ≥ university	16,195	13.79
Measured at PROBIT II		
Age, y	13,889	6.63 ± 0.27^2^
BMI, kg/m^2^	13,707	15.59 ± 1.70
Measured at PROBIT III		
Age, y	13,724	11.62 ± 0.51
SBP, mm Hg	13,702	108.08 ± 8.91
DBP, mm Hg	13,703	62.07 ± 6.85
BMI, kg/m^2^	13,671	18.14 ± 2.99
ChEAT-24 score ≥85th percentile	13,596	17.28
ChEAT-25 score ≥85th percentile	13,596	14.58
ChEAT-24 score ≥91st percentile	13,596	10.88
ChEAT-25 score ≥91st percentile	13,596	11.61
Measured at PROBIT IV		
Age, y	13,557	16.15 ± 0.54
FMI, kg/m^2^	13,396	4.08 ± 2.40
SBP, mm Hg	13,539	120.16 ± 11.24
DBP, mm Hg	13,547	68.28 ± 7.26
New-onset overweight^3^ (*n* = 11,612)^4^	772	6.65
New-onset high obesity^5^ (*n* = 13,327)^4^	198	1.49
New-onset high SBP^6^ (*n* = 13,010)^4^	524	4.03
New-onset high DBP^6^ (*n* = 13,011)^4^	530	4.07

1 Values are percentages unless otherwise specified. ChEAT, Children’s Eating Attitudes Test; DBP, diastolic blood pressure; FMI, fat mass index; PROBIT, Promotion of Breastfeeding Intervention Trial; SBP, systolic blood pressure.

2 Mean ± SD (all such values).

3 Categories of BMI for underweight, overweight, and obesity in children were defined by Wade et al. (28) and Cole et al. (38, 39) and mapped onto the WHO categories for adults. New-onset overweight was defined as those who were not overweight or obese at age 11.5 y but were overweight at age 16 y.

4 Numbers represent the numerators of derived new-onset outcomes [e.g., of the total number of children who were not obese at age 11.5 y for new-onset obesity (*n* = 13,327), 198 were obese at age 16 y]; the percentage reflects this outcome (e.g., for new-onset obesity, 1.49% of those who were not obese at age 11.5 y were obese at age 16 y).

5 New-onset obesity was defined as those children who were not obese at age 11.5 y but were obese at age 16 y.

6 Children who did not have a high SBP or DBP at age 11.5 y (<95th percentile) who went on to develop high SBP or DBP at age 16 y (≥95th percentile).

Problematic eating attitudes at age 11.5 y were positively associated with new-onset obesity (OR: 2.05; 95% CI: 1.49, 2.81) that remained after controlling for sociodemographic confounders (OR: 2.18; 95% CI: 1.58, 3.02) but not with new-onset overweight at age 16 y (**Table 2**). This association attenuated slightly after controlling for BMI at age 6.5 y (OR: 1.80; 95% CI: 1.28, 2.53).

**TABLE 2:** Associations between having a ChEAT-24 score ≥85th percentile compared with a ChEAT-24 score <85th percentile measured at age 11.5 y and cardiometabolic outcomes at age 16 y^1^

Outcome	*n*^2^	Age- and sex-adjusted model	*P* value	Confounder-adjusted model^3^	*P* value	Additionally adjusted for BMI at age 6.5 y^4^	*P* value
New-onset overweight at age 16 y^5^	9318	1.16 (0.95, 1.43)	0.15	1.18 (0.96, 1.46)	0.12	1.12 (0.90, 1.40)	0.32
New-onset obesity at age 16 y^6^	10,670	2.05 (1.49, 2.81)	<0.0001	2.18 (1.58, 3.02)	<0.0001	1.80 (1.28, 2.53)	0.001
New-onset high SBP at age 16 y^7^	10,410	1.30 (1.02, 1.64)	0.03	1.34 (1.05, 1.70)	0.02	1.14 (0.89, 1.46)	0.31
New-onset high DBP at age 16 y^7^	10,404	1.20 (0.95, 1.50)	0.12	1.25 (0.99, 1.58)	0.06	1.09 (0.86, 1.39)	0.47

1 Estimates represent the ORs (95% CIs) for each binary outcome associated with having a ChEAT-24 score ≥85th percentile (≥22.5) compared with a ChEAT-24 score <85th percentile (<22.5). ChEAT, Children’s Eating Attitudes Test; DBP, diastolic blood pressure; SBP, systolic blood pressure.

2 Total number of children with full data on each outcome and all confounders.

3 Adjusted for age at outcome measure, sex, clustering by hospital or polyclinic, treatment group, location of polyclinic site, highest household occupation, and maternal and paternal education.

4 Additionally adjusted for BMI measured at age 6.5 y.

5 Categories of BMI for underweight, overweight, and obesity in children were defined by Wade et al. (28) and Cole et al. (38, 39) and mapped onto the WHO categories for adults. New-onset overweight was defined as those who were not overweight or obese at age 11.5 y but were overweight at age 16 y.

6 New-onset obesity was defined as those children who were not obese at age 11.5 y but were obese at age 16 y.

7 Children who did not have a high SBP or DBP at age 11.5 y (<95th percentile) who went on to develop high SBP or DBP at age 16 y (≥95th percentile).

Problematic eating attitudes at age 11.5 y were positively associated with new-onset high SBP (OR: 1.30; 95% CI: 1.02, 1.64) at age 16 y (Table 2) that remained after controlling for sociodemographic confounders (OR: 1.34; 95% CI: 1.05, 1.70). A similar positive association was observed between problematic eating attitudes and new-onset high DBP at age 16 y in confounder-adjusted models (OR: 1.25; 95% CI: 0.99, 1.58). All associations with BP attenuated after controlling for BMI at age 6.5 y (Table 2).

Sex-specific results were similar to the pooled results (**Supplemental Tables 4** and **5** for females and males, respectively), with additional evidence for a positive association between problematic eating attitudes at age 11.5 y in boys and new-onset overweight at age 16 y in fully adjusted models (OR: 1.69; 95% CI: 1.24, 2.31). Most results were consistent or stronger when using a more stringent threshold for ChEAT-24 (≥91st percentile) (**Supplemental Table 6**) and a ChEAT-25 score both ≥85th percentile (**Supplemental Table 7**) and ≥91st percentile (**Supplemental Table 8**). In sensitivity analyses that used continuous outcome measures of FMI and BP at age 16 y (**Supplemental Table 9**), problematic eating attitudes were positively associated with FMI (difference in means: 0.51 kg/m^2^; 95% CI: 0.41, 0.62 kg/m^2^) and SBP (difference in means: 0.66 mm Hg; 95% CI: 0.16, 1.17 mm Hg); however, after adjusting for BMI at age 6.5 y, only the association with FMI remained (difference in mean: 0.21 kg/m^2^; 95% CI: 0.12, 0.31 kg/m^2^). After adjusting for BMI at age 11.5 y rather than at 6.5 y, the estimates of association between problematic eating attitudes at age 11.5 y and all new-onset outcomes attenuated (**Supplemental Table 10**).

## DISCUSSION

In this large prospective cohort study set in Belarus, we found that problematic eating attitudes at age 11.5 y predicted new-onset obesity, new-onset high BP, and higher FMI at age 16 y. After controlling for sociodemographic confounders and BMI, we observed that children who reported problematic eating attitudes at age 11.5 y were almost twice as likely (CIs 1.3–2.5 times more likely) to develop new-onset obesity at age 16 y. This finding suggests that problematic eating attitudes in midchildhood is linked to the later development of obesity, beyond any influence of early-childhood levels of adiposity on later eating attitudes. The observed associations with high SBP and DBP were attenuated after controlling for BMI at age 6.5 y, suggesting that adiposity in early life may underlie later problematic eating attitudes and confound the relation with subsequent adolescent BP (28, 44, 45).

Our results are consistent with previous reports that individuals with problematic eating attitudes and behaviors (including loss-of-control eating, dietary restraint, and weight control) during childhood have an increased risk of binge eating, high weight gain, and obesity later in life (18–21). For example, a study of >14,000 children aged 9–14 y reported that children who were considered dieters at baseline gained more weight over 3 y than those considered nondieters, even after adjusting for socioeconomic confounders, energy intake, physical activity, and Tanner stage of pubertal development (19). In another study of >2000 adolescents, those with an unhealthy diet or drastic measures of weight control (e.g., fasting, eating little food, skipping meals, using diet pills or food substitutes, and vomiting) at a mean age of 13 y were more likely to have increased body weight 5 y later than were those who had comparatively healthy eating behaviors (15). We are unaware of previous studies investigating problematic eating attitudes in childhood with BP in later life.

Strengths of our study include its prospective design, large sample size, high follow-up rate, and measurement of important potential confounders in early life, including repeated measurements of adiposity. However, we did not have repeated ChEAT-24 scores measured throughout childhood, which would have allowed a more convincing demonstration of the direction of association between eating attitudes and our study outcomes (43). Although we hypothesized that BMI at age 11.5 y may have mediated the association between problematic eating attitudes at 11.5 y and new-onset outcomes at age 16 y, adjusting for BMI at age 11.5 y as a confounder in the association of interest led to an attenuation of estimates. This attenuation could either have been caused by a confounding or mediation effect of BMI at age 11.5 y, but we had no way of testing this with the available data. Other limitations to our study include its possible nongeneralizablility to settings with different sociocultural and economic characteristics. Furthermore, the ChEAT questionnaire is only a proxy for problematic eating attitudes, although it has been previously validated against measures of disordered eating (24), including the Eating Disorder Examination adapted for children, revised Eating Disorder Inventory body dissatisfaction subscale, Rosenburg Self-Esteem Scale, Children’s Depression Inventory, and the related Eating Attitudes Test predicts clinically important eating concerns, weight preoccupation, or disordered eating in adults (34).

These relatively novel findings have potentially important public health and clinical implications that are 2-fold: first, the immediate impact should be to make health professionals aware that problematic eating attitudes in children may contribute to later obesity; second, in the longer term, the findings motivate the development and testing (with the use of randomized controlled trials) of interventions to prevent subsequent overweight and obesity. Previous literature has suggested that clinical and earlier group-based interventions may be effective in preventing problematic eating attitudes. Clinically diagnosed eating disorders may require more specialized mental health interventions (13).

We conclude that problematic eating attitudes observed in a large cohort in midchildhood were prospectively associated with the subsequent development of obesity. Therefore, early identification and management of problematic eating attitudes in early life may be an important factor in reducing the obesity epidemic in later adolescence. Independent replication in large-scale prospective studies is required to fully understand the complex association between eating attitudes and cardiometabolic health.

## Footnotes

12 Abbreviations used: BP, blood pressure; ChEAT, Children’s Eating Attitudes Test; DBP, diastolic blood pressure; FMI, fat mass index; PROBIT, Promotion of Breastfeeding Intervention Trial; SBP, systolic blood pressure.
